# Hypothalamic-pituitary-gonadal axis hormones and cortisol in both menstrual phases of women with chronic fatigue syndrome and effect of depressive mood on these hormones

**DOI:** 10.1186/1471-2474-5-47

**Published:** 2004-12-08

**Authors:** Remzi Cevik, Ali Gur, Suat Acar, Kemal Nas, Ayşegül Jale Sarac

**Affiliations:** 1 Physical Medicine and Rehabilitation, School of Medicine, Dicle University, Diyarbakir, Turkey

## Abstract

**Background:**

Chronic fatigue syndrome (CFS) is a disease which defined as medically unexplained, disabling fatigue of 6 months or more duration and often accompanied by several of a long list of physical complaints. We aimed to investigate abnormalities of hypothalamic-pituitary-gonadal (HPG) axis hormones and cortisol concentrations in premenopausal women with CFS and find out effects of depression rate on these hormones.

**Methods:**

We examined follicle stimulating hormone (FSH), luteinizing hormone (LH), estradiol, progesterone and cortisol concentrations in 43 premenopausal women (mean age: 32.86 ± 7.11) with CFS and compared matched 35 healthy controls (mean age: 31.14 ± 6.19). Patients were divided according to menstrual cycle phases (follicular and luteal) and compared with matched phase controls. Depression rate was assessed by Beck Depression Inventory (BDI), and patients with high BDI scores were compared to patients with low BDI scores.

**Results:**

There were no significant differences in FSH, LH, estradiol and progesterone levels in both of menstrual phases of patients versus controls. Cortisol levels were significantly lower in patients compared to controls. There were no significant differences in all hormone levels in patients with high depression scores versus patients with low depression scores.

**Conclusion:**

In spite of high depression rate, low cortisol concentration and normal HPG axis hormones of both menstrual phases are detected in premenopausal women with CFS. There is no differentiation between patients with high and low depression rate in all hormone levels. Depression condition of CFS may be different from classical depression and evaluation of HPG and HPA axis should be performed for understanding of pathophysiology of CFS and planning of treatment.

## Background

Chronic fatigue syndrome (CFS) is a clinical presentation that primarily affects women and characterized by severe disabling fatigue, and other symptoms; including musculoskeletal pain, sleep disturbance, impaired concentration, and headaches, in the absence of organic illness or severe psychiatric disorder that would explain the fatigue. For diagnosis of CFS operational criteria have been developed by Centers for Disease Control (CDC) [1]. Although the cause CFS is poorly understood, there are some causative theories for underlying conditions. Hypotheses about its etiology have included viral infections, immune dysregulation and abnormal endocrine function among others [2].

It has been reported that onset of CFS mostly following a significant stressor, most frequently a viral infection, and the course of the syndrome remits and relapses with occurrence of physical and psychological stressors [3]. Stress is known to interfere with the menstrual cycle and may lead to chronic anovulation and amenorrhea [4]. This generally thought to be caused by a decrease in the activity of hypothalamic gonadotropin releasing hormone (GnRH) pulse generator with subsequent inhibition of the pituitary-gonadal axis [5,6]. Stress-induced activation of hypothalamic-pituitary-adrenal (HPA) hormonal axis plays an important role in suppressing the HPG axis [7]. Infusion of corticotropin releasing hormone (CRH) into the cerebral ventricles leads to inhibition of LH secretion in primates [8]. CRH antagonism has also been shown to prevent the inhibitory effect of stress on the HPG axis in the rodent and in the monkey [9]. Women with hypothalamic amenorrhea have higher basal cortisol levels and blunted cortisol response to exogenous administration of CRH, suggesting that the increase in cortisol secretion may reflect increased endogenous CRH activity [10].

Pertuberations of HPA axis function have been described in CFS [11,12]. Studies of the HPA axis in CFS show a mild hypocortisolism of central origin, in contrast to the hypercortisolism of major depression [13,14]. There are similarites between onset, course, and clinical syndromes of CFS and glucocorticoid defficiency states. Clinical syndromes of CFS and Addison's disease share many common features: one of the principal clinical features of Addison's is fatigue, the core feature of CFS. The other common symptoms of CFS include arthralgia, myalgia, adenopathy, exacerbation of allergic responses, intermittent fever, postexertional fatigue, and depressed mood. These symptoms can also be experienced by those withdrawing from hypercortisolaemic states [15].

CFS occurs more commonly in women [16]. It was suggested that alterations in reproductive hormone levels might be involved in the pathoetiology of CFS [17]. There has been reported that this condition may be due to estrogen deficiency and reflect underactivity of the HPG hormonal axis [18]. The GnRH secretion from hypothalamus drives secretion of LH and FSH from pituitary gonadotropes [19]. FSH and LH govern the cyclical secretion of estradiol and progesterone over the course of menstrual cycle. The pulsatile pattern of GnRH secretion is critical for the control of serum LH, FSH, and ovulation.

Interaction between HPA and HPG axes in stress-induced amenorrhea suggests that there may be perturbation of these axes in CFS. One important confound is co-morbid depressive illness, present in approximately 50% of CFS patients [20]. Relation between depressive mood and these axes has been more investigated yet. This relationship may contribute to clarification of pathoetiology of CFS and to describe treatment strategy of this complex syndrome.

In this study, we aimed to investigate main hormones of HPG and HPA axes; FSH, LH, estradiol, progesterone and cortisol levels in premenopausal women with CFS. Furthermore to find out relationship between these hormones and depressive mood with comparing patients with high and low depression scores.

## Methods

### Subjects

A total of 43 premenopausal women diagnosed as CFS, according to the international CFS definition criteria [1], were recruited from outpatient clinic of Physical Medicine and Rehabilitation Department of Dicle University (Diyarbakýr, Turkey) for this study. Fatigue assessment was done according to CDC criteria [1]. Fatigue characteristics are persistent or recurrent lasting at least 6 months; recent and/or well defined onset; not secondary to excessive physical activity, or any organic or psychiatric disorders; not resolved by rest; and inducing important reduction of previous levels of physical and mental activities. Thirty five age matched demographically similar healthy premonopausal women were also selected as controls. Ethic committee of Dicle University hospital approved the study, and all subjects voluntarily agreed to participate. All patients underwent medical screening that included physical examination and relevant investigation, with a minimum of urine analysis, full blood count, measurement of urea, electrolytes, and erythrocyte sedimentation rate, and test for thyroid and liver function. All patients and controls were evaluated by structered psychiatric interview to exclude any additional psychiatric disorder prior to inclusion in the study. Depression rate was assessed by Beck Depression Inventory (BDI) in all patients and controls. Patients with CFS were divided to two groups according to the BDI higher and lower than 17 scores. All prescription medications, included psychoactive and non-prescription medications, vitamins, and herbal remedies were tapered and then stopped at least 2 weeks prior to study [17,21]. All subjects and controls had no frank hypocortisolism on endocrine assessment. No patients and controls had received any oral or intraarticular corticosteroid therapy during the three months preceding the study, or had received exogenous estrogens, progesterone, or any other drugs affecting sex or cortisol hormones metabolism. The other exclusion critetria [1] were: active, unresolved, or suspected disease likely to cause fatigue; alcohol or other substance abuse within 2 years prior to onset of the chronic fatigue and any time afterward; and a body mass index ≥45. All subjects and controls were undergoing normal menstrual cycles and were not taking contraceptive pill. Subjects and controls were all studied during the follicular (n: 28 with CFS and n: 23 controls) and luteal (n: 15 with CFS and n: 12 controls) phases of menstrual cycle.

### Procedures and Hormone Assays

Blood samples were collected in the early morning (8.30–10.30 AM) after an all night fast and plasma was separated immediately by centrifugation; then sera obtained were stored at -20°C until hormonal assaying. All hormone values assayed by "Electro Chemil Luminescence Immunassay (ECLIA)" (Roche, 1010/1020 Elecsys Systems Immunoassay) method. Serum concentrations of follicle stimulating hormone (FSH, normal values 3.3 to 11.3 mIU/mL for follicular phase and 1.8 to 8.2 mIU/mL for lueal phase), luteinizing hormone (LH, normal values 2.4 to 12.6 mIU/mL for follicular phase and 1.0 to 11.4 mIU/mL for luteal phase), estradiol (E_2_, normal values 24.5 to 195 pg/mL for follicular phase and 40 to 261 pg/mL for luteal phase), progesterone (normal values 0–1.6 ng/mL for follicular phase and 1.1 to 21 ng/mL for luteal phase), and cortisol (normal values 6.2 to 19.4 μgr/dl) were evaluated in all patients and controls in both of menstrual periods.

### Statistical analysis

were done by SPSS 8.0 PC program. Results were expressed as means ± SD (standard deviation). Statistical significances were tested using the independent student's t test for women with CFS and control group comparisons. Mann Whitney U test was used for comparison of hormonal data of women in both phases of menstrual cycle, and patients with high and low BDI scores groups comparison. The level of statistical significance was set at a two-tailed p-value of 0.05.

## Results

All patients with CFS and healthy controls were reproductive premenopoausal women; and mean ages were 32.76 ± 7.07 and 31.14 ± 6.19 respectively for both groups. All of the patients had debilitating clinically evaluated and medically unexplained fatingue that does not resolve with bed rest and severe enough to significantly reduce daily activity for at least 6 months. The other clinical findings of patients with CFS were summarized in Table 1.

There wasn't significant differentiation between ages and BMI of groups (p > 0.05). There were no significant differences between the means of FSH, LH, progesterone and estradiol in all CFS patients compared to all controls (p > 0.05). Mean concentrations of cortisol were significantly lower in all CFS patients than all controls (p < 0.001) (Table 2).

There were no significant differences between the levels of FSH, LH, progesterone and estradiol in CFS patients compared to controls in follicular phase (p > 0.05). Mean concentrations of cortisol were significantly lower in CFS patients than controls in follicular phase (p = 0.001) (Table 3).

There were no significant differences between the levels of FSH, LH, progesterone and estradiol in CFS patients compared to controls in luteal phase (p > 0.05). Mean concentrations of cortisol were significantly lower in CFS patients than controls in luteal phase (p < 0.05) (Table 4).

Fifty patients (69.76%) with CFS had ≥17 BDI scores. Mean cortisol concentrations of patients with BDI scores ≥17 were higher than patients with BDI scores <17 but differentiation wasn't significant. There were no significant differences between these patients groups in HPG axis hormone levels (Table 5).

## Discussion

In this study, reproductive HPG axis hormone levels demonstrated no significant differences in women with CFS from controls during follicular and luteal phases. These findings are in agreement with Korszun et al. [17] who reported data from 9 premenopausal women with fibromyalgia and 8 with CFS. They showed no significant differentiations of reproductive axis function in both of patients groups in estrogen and progesterone levels, as well as LH pulsatility during the follicular phase. However, Studd and Panay [18] reported data from 28 premenopausal women with CFS 87 and of these, 25% showed low plasma estradiol concentrations. The authors reported that CFS may represent an hypoestrogenic state and recommend the use of hormone replacement therapy for women with CFS. In addition, they claim that 80% of patients improved after treatment of estradiol patches and cyclical progestagens.

Chronic fatigue syndrome is generally accepted stress related disease and disfunction of HPA axis has been reported in this syndrome [22]. In this study, cortisol levels were lower in women with CFS compared to healthy controls. Some sudies of HPA in CFS show a mild hypocortisolism of central origin in contrast to hypercortisolism of major depression [13,14]. In an early study of the HPA axis in patients with CFS Demitract et al. [22] reported low 24-hour urine free cortisol compared with that of control subjects. Baseline evening plasma corticotropin levels were elevated and cortisol levels were depressed. Significantly lower baseline cortisol levels was reported in an earlier study [23]. Despite these findings, the majority of further studies have failed to replicate this. Differentiations of studies methodology and sample characteristics may explain the variety of results.

High circulating cortisol is well replicated finding in major depression [24] and so presence of depression makes the cortisol findings more difficult to interpret. Significantly raised baseline cortisol levels in subjects with CFS studied by Wood et al. [25] was explained by their high BDI scores [20]. In this study, high BDI scores (≥17) were detected in 69.76% of patients with CFS. There were no significant high level of cortisol and HPG axis hormone concentrations in patients with high BDI score compared to patients with low BDI scores (<17). Scott and Dinan [14] reported finding of low urine free cortisol in patients with CFS compared with healthy controls. In addition, there was no difference between depressed and non depressed patients with CFS. These findings are in agreement with our study. In another study [26] was reported blunted corticotropin and cortisol in response to administration of ovine CRH without differences in basal levels.

Studies in primates have demonstrated that intracerebroventricular infusion of CRH as well as proinflammatory cytokines such as interleukin-1 can decrease LH secretion [27,28]. Stress induced (hypothalamic) amenorrhea, as well as exercise-induced amenorrhea and anorexia nervosa, activate the HPA axis, increasing cortisol secretion and decreasing corticotropin or cortisol response to exogenous CRH [29-31]. These HPA axis abnormalities are similar to those seen in depression, suggesting that activation of the HPA axis may be linked to inhibition of the HPG axis. Young et al. [32] found %30 lower plasma estradiol level in women with depression than controls in the follicular phase. The half-life LH was significantly shorter in women with depression than controls during both of the follicular and luteal phases. The other reproductive hormones were normal in women with depression compared to control women in both the phases of menstrual cycle.

In this study was found significantly lower circulating cortisol levels in patients with CFS in contrast to high BDI scores. However, there was no significant differentiation in cortisol levels between the patients with low and high depression scores. This is in contradiction to those hypercortisolism of classical major depression and stress condition. More recent studies support this contradicton [21,33]. In recent years, however, it has become increasingly apparent that deppression is a heterogenous condition from both a psychological and a physiological perspective [34]. Moreover, decreased HPA axis activity was reported in some stress related states such as CFS, atypical and seasonal depression [35]. These results suggest that depression condition which is seen in CFS may be different from classical depression. There may be overlapping between symptoms of CFS and those depressive subtypes or reactive form of depression in CFS. This condition may explaine both hypocortisolism in patients with CFS and the lack of HPG axis hormone abnormalities in this study.

Determining single basal levels of HPA and HPG axes do not reflect activity of these axes entirely. Dynamic test indicates differences in function of these axes. We did not carry out dynamic characteristics of these axes. This point is limit of our study.

In conclusion, we detected low cortisol levels in patients with CFS in spite of their high depressive mood rate. However, in this study, we were unable to describe HPG axis hormone abnormalities in both menstrual phases. Hypocortisolism may be a biological factor that contributes fatigue chronicity and the reason of normality in HPG axis. Depressive mood of chronic fatigue syndrome may be different from classical depression. There are need to carry out future controlled and larger clinical trials to clarify these matters.

## Pre-publication history

The pre-publication history for this paper can be accessed here:



## Figures and Tables

**Table 1:** Clinical findings of 43 patients with CFS

	(N)	(%)
**Fatigue**	43	100
**Sore Throat**	32	74.42
**Arthralgıa**	34	79.07
**Myalgıa**	39	90.7
**Muscle Weaknes**	7	16.27
**Fever**	24	55.8
**Fharyngitis**	32	74.42
**Lenfadenopathy**	31	72.1
**Headache**	23	53.5
**Neuropsychologic findings**	43	100
**Sleep Disturbance**	31	72.1

**Table 2:** HPG axis hormonal data and cortisol levels in all patients and controls

	Patients (n = 43)	Controls (n = 35)	T	p
Age	32.76 ± 7.07	31.14 ± 6.19	1.06	0.290
BMI	24.47 ± 4.37	24.63 ± 4.39	-0.147	0.883
BDI	21.83 ± 8.49	11.6 ± 3.45	6.68	0.000
FSH	6.57 ± 4.87	6.52 ± 7.45	0.034	0.973
LH	6.82 ± 5.72	7.09 ± 6.04	-0.202	0.841
Progesterone	2.23 ± 3.14	3.15 ± 4.21	-1.105	0.273
Estradiol	111.54 ± 176.83	89.08 ± 55.37	0.722	0.472
Cortisol	10.33 ± 4.55	15.11 ± 6.95	-3.64	0.000

**Table 3:** HPG axis hormonal data and cortisol levels in patients and controls in follicular phase

	Patients (n = 28)	Controls (n = 23)	T	P
Age	32.71 ± 7.43	30.74 ± 6.39	-1.17	0.240
BDI	22.85 ± 9.04	12.3 ± 3.26	-4.71	0.000
FSH	6.73 ± 5.32	7.91 ± 8.84	-0.142	0.88
LH	7.6 ± 6.37	8.34 ± 6.85	-0.58	0.55
Progesterone	1.96 ± 2.95	2.10 ± 3.56	-0.22	0.83
Estradiol	129.65 ± 217.05	89.66 ± 58.21	-0.21	0.83
Cortisol	10.12 ± 4.46	15.26 ± 7.17	-3.25	0.001

**Table 4:** HPG axis hormonal data and cortisol levels in patients and controls in luteal phase

	Patients (n = 15)	Controls (n = 12)	T	P
Age	32.86 ± 6.58	31.91 ± 5.99	-0.34	0.75
BDI	19.93 ± 7.26	10.25 ± 3.57	-3.30	0.000
FSH	6.26 ± 4.07	3.87 ± 1.99	-1.66	0.103
LH	5.36 ± 4.03	4.69 ± 3.05	-0.53	0.61
Progesterone	2.72 ± 3.5	5.19 ± 4.76	-1.415	0.167
Estradiol	77.73 ± 32.61	87.97 ± 51.94	-0.293	0.792
Cortisol	10.33 ± 4.3	14.81 ± 6.81	-2.001	0.047

**Table 5:** Cortisol levels in women with CFS according to BDI higher and lower than 17 scores

	BDI ≥17 scores (n = 30)	BDI scores <17 (n = 13)	T	P
Age	33.76 ± 6.89	30.46 ± 7.20	-1.52	0.130
BDI	25.93 ± 6.7	12.38 ± 2.29	-5.17	0.000
FSH	6.62 ± 5.36	6.46 ± 3.72	-0.212	0.84
LH	6.71 ± 6.25	7.08 ± 4.5	-0.80	0.38
Progesterone	2.08 ± 2.92	2.56 ± 3.69	-0.42	0.68
Estradiol	127.46 ± 206.32	74.79 ± 66.81	-1.45	0.151
Cortisol	10.37 ± 4.65	9.78 ± 3.8	-0.42	0.68
